# Beneficial effect of Xuebijing against *Pseudomonas aeruginosa* infection in *Caenorhabditis elegans*

**DOI:** 10.3389/fphar.2022.949608

**Published:** 2022-08-31

**Authors:** Le Zhang, Yuxing Wang, Chang Cao, Yike Zhu, Wei Huang, Yi Yang, Haibo Qiu, Songqiao Liu, Dayong Wang

**Affiliations:** ^1^ Jiangsu Provincial Key Laboratory of Critical Care Medicine, School of Medicine, Southeast University, Nanjing, China; ^2^ Department of Critical Care Medicine, Zhongda Hospital, Nanjing, China

**Keywords:** Xuebijing, bacterial infection, anti-infection, pharmacological mechanism, *C. elegans*

## Abstract

In the clinical intensive care units (ICU), the traditional Chinese medicine (TCM) formulation of Xuebijing has been frequently used for treating sepsis. Nevertheless, the underlying pharmacological mechanisms of Xuebijing remain largely unclear. *Caenorhabditis elegans* is an important experimental host for bacterial infections. Using *C. elegans* as an animal model, we here examined the potential of Xuebijing treatment against bacterial infection and the underlying mechanisms. Xuebijing treatment could inhibit the reduction tendency of lifespan caused by *Pseudomonas aeruginosa* infection. For the cellular mechanisms of this antibacterial infection property, we found that Xuebijing treatment rescued *C. elegans* lifespan to be against *P. aeruginosa* infection by inhibiting *Pseudomonas* colonization in the intestinal lumen. Meanwhile, the increase in the expression of antimicrobial genes induced by *Pseudomonas* infection was also suppressed by Xuebijing treatment. Moreover, the beneficial effect of Xuebijing against *Pseudomonas* infection depended on insulin, p38 MAPK, Wnt, DBL-1/TGF-β, ELT-2, and programmed cell death (PCD)-related signals. Although Xuebijing did not show obvious antibacterial activity, Xuebijing (100%) treatment could inhibit the *Pseudomonas* biofilm formation and decrease the expression of virulence genes (*lasA*, *lasB*, *rhlA*, *rhlC*, *phzA*, *phzM*, *phzH*, and *phzS*) and quorum sensing (QS)-related genes (*lasI*, *lasR*, *rhlI*, *rhlR, pqsA*, and *pqsR*). Our results support the potential role of Xuebijing treatment against bacterial infection in hosts.

## Introduction

Sepsis is a life-threatening illness with multiple organ failure induced by severe pathogenic infection (van der Poll et al., 2017; Huang et al., 2019). In the intensive care units (ICU), sepsis has gradually become a major cause of death. During the sepsis management, the traditional Chinese medicine (TCM) application has become a growing trend (Fan et al., 2020). The formulation of Xuebijing is one of those TCMs to be used as a critical care drug for sepsis treatment (Mousavi et al., 2016). In the clinical ICU, it has been shown that Xuebijing can provide beneficial effects for patients from complications of bacterial pneumonia or sepsis (Hou et al., 2015; Zheng et al., 2018). In the ICU, Xuebijing integrated with Western medicine was also effective to treat severe cases of viral infections, such as COVID-19 infection (Ni et al., 2020; Huang et al., 2021).

Xuebijing injection contains extracts from 5 Chinese medicines, which was approved for sepsis treatment from 2004 with the anticipated efficacy and safety (Li et al., 2018). Xuebijing could balance the immune responses to pathogenic infection and thus strengthen the body resistance (Fan et al., 2020). During the treatment of sepsis, Xuebijing has shown the antioxidant, vascular endothelium protection, and anti-inflammatory effects (Li et al., 2021). Moreover, in sepsis patients, Xuebijing might improve blood circulation *via* anti-coagulation (Hou et al., 2015). Nevertheless, the pharmacological effects and the related mechanisms of Xuebijing therapy remain still largely unclear.

As a model animal, the genetic behavior of pathogenic infection can be conveniently traced in *Caenorhabditis elegans* (Balla and Troemel, 2013). In their natural habitat, *C. elegans* will meet different microbes, including bacterial pathogens (Kim and Ausubel, 2005). In response to pathogens, innate immunity will be activated in *C. elegans*, which guarantees the animals to survive long enough and to reproduce during the evolution (Martineau et al., 2021). The innate immunity is often triggered within cells of primary targeted organs, such as the epidermis and the intestine (Taffoni and Pujol, 2015; Kim and Ewbank, 2018). Upon pathogenic infection, *C. elegans* will secret antimicrobial proteins to kill pathogens (Engelmann and Pujol, 2010; Zhi et al., 2017a). Moreover, some signaling pathways [such as p38 MAPK, insulin, Wnt, DBL-1/TGF-β, ELT-2, and programmed cell death (PCD) signaling pathways] play an important role in regulating innate immunity in *C. elegans* (Kurz and Tan, 2004; Irazoqui et al., 2008; Arvanitis et al., 2013; Head et al., 2017; Yu et al., 2018). Therefore, *C. elegans* can provide a powerful model for studying the host–pathogen interactions.

*C. elegans* has the properties of short lifespan, short life cycle, and low cost for maintenance. *C. elegans* has become a useful animal model for high-throughput screening and pharmacological study of compounds against several diseases, such as pathogenic infection and neurodegeneration (Rand and Johnson, 1995; Parker et al., 2004; Giunti et al., 2021). *C. elegans* can be used to screen small molecules with anti-infective and host protection potentials by boosting innate immune responses (Peterson and Pukkila-Worley, 2018). That is, *C. elegans* is also helpful to identify preclinical drugs with antibacterial and antifungal infection functions (Anastassopoulou et al., 2011; Kim et al., 2017).

*C. elegans* has been frequently employed as a host to determine the mechanisms of *Pseudomonas aeruginosa* infection (Tan and Ausubel, 2000). In this study, *C. elegans* was used as a host, and *P. aeruginosa* was selected as a bacterial pathogen. We aimed to determine the possible potential of Xuebijing treatment against bacterial infection. Moreover, the underlying mechanism for this beneficial effect of Xuebijing was examined. Our results are helpful for further understanding the pharmacological effects and mechanisms of Xuebijing therapy.

## Methods

### Xuebijing

Xuebijing is a sterile and nonpyrogenic injection for the intravenous administration. Xuebijing is manufactured and provided by Tianjin Chase Sun Pharmaceutical Co., Ltd. (No. Z20040033). Xuebijing is approved by China Food and Drug Administration (CFDA) for sepsis treatment (ratification number, GuoYaoZhunZi-Z20039833). Xuebijing injection is prepared from a combination of *Carthamus tinctorius* L. (Carthami Flos, Honghua), *Paeonia lactiflora* Pall. (Paeoniae Radix Rubra, Chishao), *Ligusticum chuanxiong* Hort. (Chuanxiong Rhizoma, Chuanxiong), *Salvia miltiorrhiza* Bge. (Salviae Miltiorrhizae Radix Et Rhizoma, Danshen), and *Angelica sinensis* (Oliv.) Diels (Angelicae Sinensis Radix, Danggui), yielding a botanical drug-to-injection ratio of 1:2 (*V*/*V*). The ingredients from *Carthamus tinctorius flowers* were extracted with ethanol, then with water. The ingredients from the other botanical drugs were extracted with water. Finally, Xuebijing is standardized to contain 200–500 μg/mL hydroxysafflor yellow A and 1000–1700 μg/mL paeoniflorin (Huang et al., 2011; Chen et al., 2016; Li et al., 2016). The pH value of Xuebijing injection is 5.5.

The fingerprint of Xuebijing determined by the method of reverse-phase high-performance liquid chromatography (RP-HPLC) was provided in Supplementary Figure S1. Among the used standards, the protocatechuic aldehyde is the marker compound of Danshen, hydroxysafflor yellow A is the marker compound of Honghua, paeoniflorin is the marker compound of Chishao, and ferulic acid and senkyunolide are marker compounds of Chuanxiong and Danggui (Supplementary Figure S1).

### *C. elegans* strain and culture

The used strain was wild-type N2, which was normally cultured on nematode growth medium (NGM) plates as described (Brenner, 1974). The NGM plates were seeded with *Escherichia coli* OP50 as the food source of nematodes. To perform bacterial infection and Xuebijing treatment, synchronized young-adult nematodes were needed to be prepared. Gravid hermaphrodites were treated by bleaching solution (0.45 M NaOH and 2% HOCl) to release enough eggs (Zhao Y.-Y. et al., 2022). The obtained eggs were placed in new NGM plates and then developed into synchronized young adults.

### Bacterial preparation

*P*. *aeruginosa* PA14 was cultured in the Luria broth. *P*. *aeruginosa* PA14 was seeded on the modified NGM killing plate containing 0.35% peptone. The killing plates were incubated at 37°C for 24 h and further at 25°C for 24 h.

### Bacterial infection

Young adults were transferred onto full-lawn PA14 killing plates for bacterial infection (Zhi et al., 2017b). Fifty animals were added to each killing plate. The bacterial infection was performed at 25°C for 24 h. Three replicates were carried out.

### Pharmacological post-treatment

**FIGURE 1:**
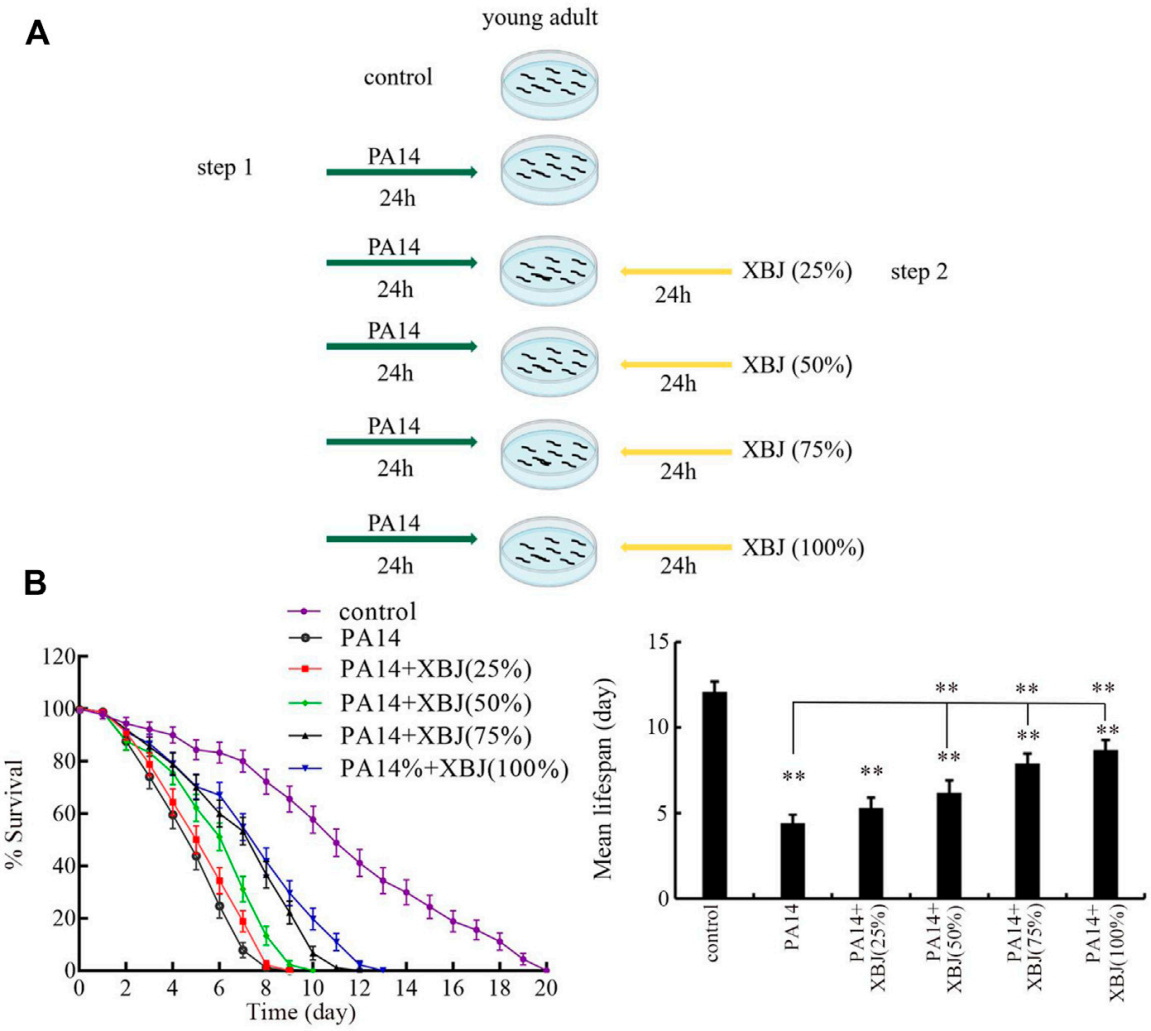
Effect of Xuebijing post-treatment against *P*. *aeruginosa* infection. **(A)** Diagram showing the method for pharmacological post-treatment of Xuebijing after *P*. *aeruginosa* infection. **(B)** Effect of Xuebijing post-treatment on lifespan of nematodes after *P*. *aeruginosa* infection. XBJ, Xuebijing. **p < 0.01 vs. control (if not specially indicated).

After the exposure of young adults to PA14 for 24, the nematodes were further treated with Xuebijing for 24 h (Figure 1A). The 25%, 50%, 75%, and 100% (the original Xuebijing solution) Xuebijing were used for pharmacological treatment. Experiments were carried out in triplicate.

### Lifespan assay

*C. elegans* lifespan was examined as described (Li et al., 2020). After bacterial infection and Xuebijing treatment, the nematodes were transferred onto normal NGM plates. To exclude the effect from offspring, the examined nematodes were transferred daily onto new NGM plates. The nematodes were scored every day. Nematodes were considered as dead if no response could be found after prodding with a platinum wire. The mean lifespan refers to the day at which 50% animals survived. Three replicates were performed. Fifty nematodes were tested for lifespan assay. The statistical significance between lifespan curves was determined by the Kaplan–Meier survival analysis, followed by the log-rank test.

### Analysis of PA14 accumulation in the intestinal lumen

PA14 colony-forming unit (CFU) in the body of nematodes was analyzed as described (Zhi et al., 2017b). After PA14 infection and Xuebijing treatment, the nematodes were first transferred onto M9 solution containing 25 mM levamisole in order to paralyze the animals and stop their pharyngeal pumping. The nematodes were then transferred onto a plate containing 1 mg/mL gentamicin and 1 mg/mL ampicillin to treat for 30 min in order to eliminate PA14 on the surface of the body. After that, each group of fifty animals was lysed using a motorized pestle, serially diluted by M9 buffer, and transferred onto a Luria–Bertani (LB) plate containing 100 μg/mL rifampicin. After incubation at 37°C overnight, the PA14 colony number was counted. Ten replicates were performed.

To determine the PA14 accumulation in the intestinal lumen, we also examined the PA14::GFP accumulation. The data were expressed as the relative fluorescence intensity of PA14::GFP in the intestinal lumen after normalization to intestinal autofluorescence. Fifty nematodes were tested for each group. Three replicates were carried out.

### Quantitative Real-Time polymerase chain reaction

Total RNAs of nematodes and PA14 were extracted using TRIzol reagent according to the manufacturer’s instruction. The RNA quality was evaluated according to OD260/280 ratio in NanoDrop One. A gradient MasterCycler (Eppendorf, United States) was used for cDNA synthesis. SYBR Green Master Mix was used for qRT-PCR in an ABI 7500 real-time PCR system. A comparative CT (ΔΔ CT) method was used, and the expression of examined genes was normalized with the expression of the reference gene (*tba-1* or *pvdQ*) (Yang et al., 2021a; Yang D. et al., 2021). Three replicates were performed. Primer information is shown in Supplementary Table S1, S2.

### RNA interference

In order to inhibit gene expression, RNAi was carried out by feeding the nematodes with *E. coli* HT115 expressing a certain gene (Yang et al., 2021b). In this study, the RNAi was performed after PA14 infection. On RNAi plates, the 100% Xuebijing was used for pharmacological treatment. *E. coli* HT115 expressing empty vector L4440 was used as the control (Zhang et al., 2022). RNAi efficiency was evaluated using qRT-PCR (Supplementary Figure S2).

### Analysis of the antibacterial activity of Xuebijing

1) Time–kill assay. PA14 cells cultured overnight were centrifuged and dispensed into culture tubes containing 75% or 100% Xuebijing in a volume of 5 mL. After that, PA14 cells were cultured at 35°C. Colony counts were analyzed on a LB plate at 0, 6, 12, 18, and 24 h. The ampicillin (1 μg/mL) was used as a positive control. Experiments were performed in triplicate.
2) Agar diffusion assay. After concentration by centrifugation and washing using PBS buffer, approximately 10^7^ PA14 cells/mL were inoculated in a liquid LB medium. After that, 10 mL suspensions were transferred onto a LB agar plate. Xuebijing (75% and 100%) was added onto filter disks (diameter, 6 mm) and placed on an agar surface. The plate was incubated at 35°C for 48 h. The ampicillin was used as a positive control.

### Analysis of bacterial biofilm formation

The bacterial biofilm formation was analyzed using the crystal violet method (Lee et al., 2011). The PA14 culture (approximately 5 × 10^5^ CFU/mL) was taken in a 96-well plate to incubate with 100% Xuebijing at 37°C for 36 h. After the incubation, the biofilm was washed with PBS buffer, fixed with methanol for 15 min, and stained with 0.1% crystal violet for 15 min. The absorbance was also measured to quantify biofilm formation at 595 nm. Three replicates were performed.

### Data analysis

Data are presented as means ± SD. The SPSS 12.0 software was used for statistical analysis. Differences between the different groups were analyzed by analysis of variance (ANOVA). A probability level of 0.01 was considered statistically significant.

## Results

### Effect of Xuebijing treatment against bacterial infection

After PA14 infection, the sharply reduced lifespan was observed from day 2 to day 8 (Figure 1B). Treatment with 25% Xuebijing did not obviously affect the reduction in lifespan observed in PA14-infected nematodes (Figure 1B; Supplementary Table S3). Different from this, treatment with Xuebijing (50–100%) could significantly suppress the lifespan reduction observed in PA14-infected nematodes, and this beneficial effect of Xuebijing treatment was dose-dependent (Figure 1B; Supplementary Table S3). Therefore, Xuebijing treatment had the beneficial effect against bacterial infection.

### Effect of Xuebijing treatment against bacterial colony formation in nematodes

**FIGURE 2:**
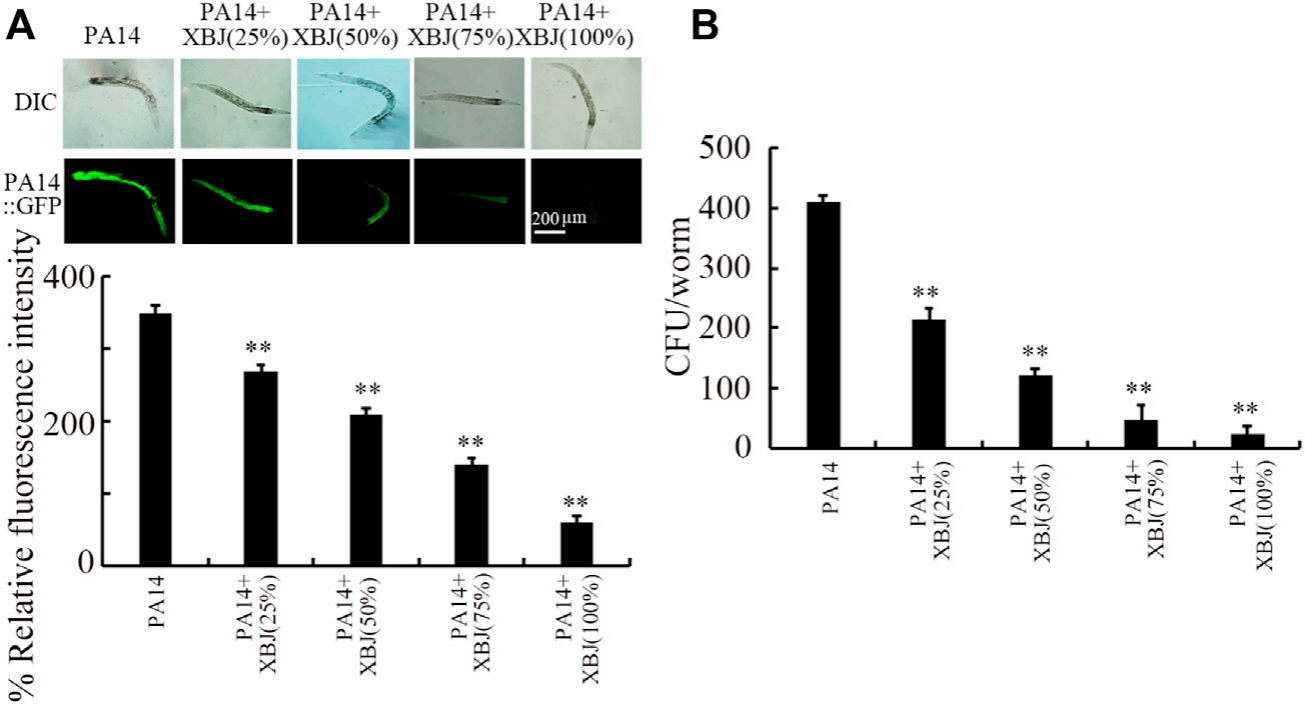
Effect of Xuebijing post-treatment on PA14:GFP **(A)** and CFU **(B)** in *P*. *aeruginosa*-infected nematodes. XBJ, Xuebijing. **p < 0.01 vs. control.

To examine the underlying mechanisms for the beneficial effect of Xuebijing treatment against bacterial infection, we next analyzed the colony formation of PA14 in the intestine. After infection, a pronounced PA14::GFP accumulation was observed in the intestinal lumen (Figure 2A). Similarly, a high level of PA14 CFU was detected in the intestine (Figure 2B). Treatment with Xuebijing (25%–100%) could significantly suppress PA14::GFP accumulation in the intestine and inhibit the formation of high intestinal CFU (Figures 2A,B). Among the examined concentrations, the 100% Xuebijing exhibited the most beneficial effect against the bacterial colony formation in the intestine of nematodes.

### Effect of Xuebijing treatment on innate immune response after bacterial infection

**FIGURE 3:**
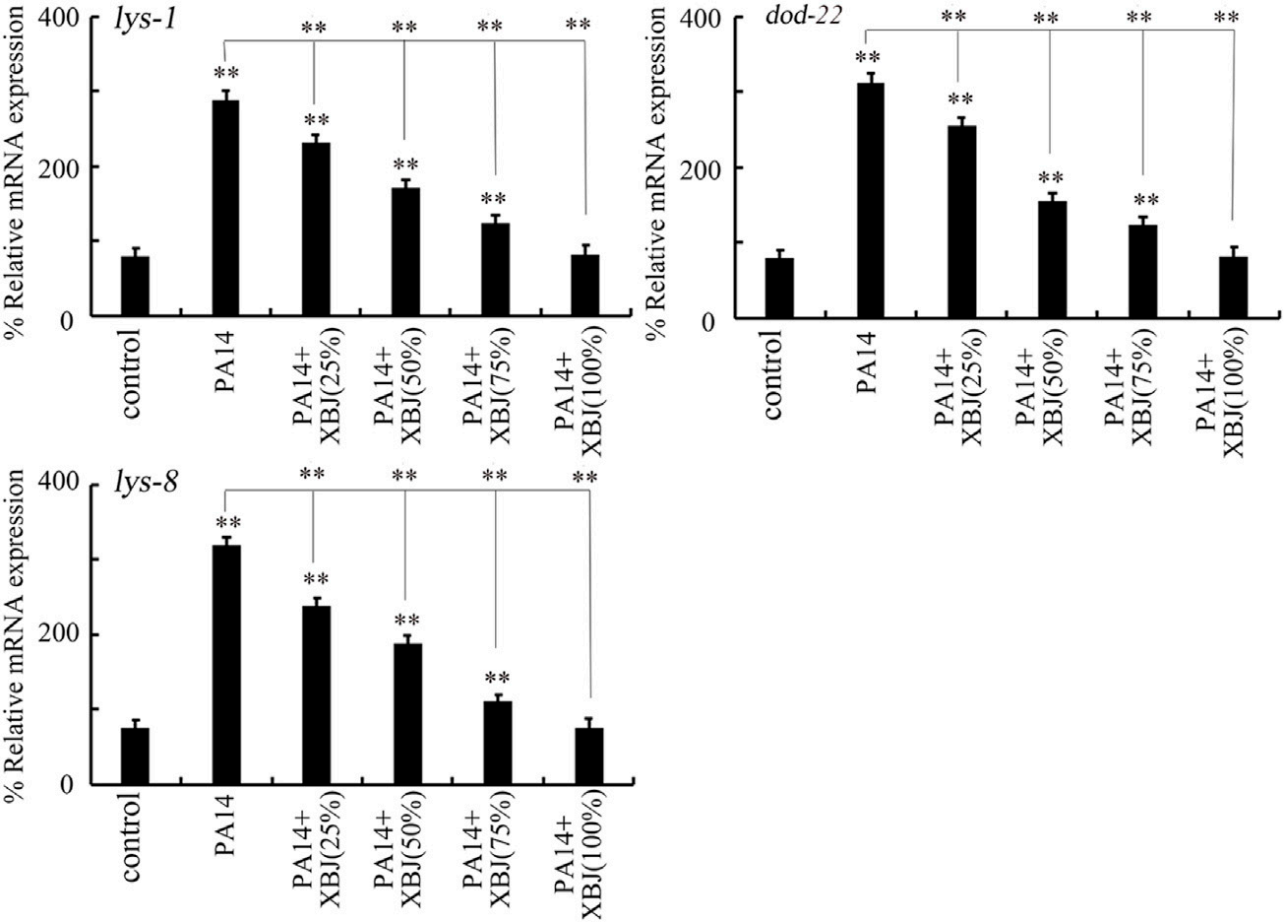
Effect of Xuebijing post-treatment on antimicrobial gene expression in *P*. *aeruginosa*-infected nematodes. XBJ, Xuebijing. **p < 0.01 vs. control (if not specially indicated).

The *lys-1*, *dod-22*, and *lys-8* were selected as intestinal antimicrobial genes in response to PA14 infection (Zhi et al., 2017b). PA14 infection induced a noticeable increase in the expression of these 3 antimicrobial genes (Figure 3). After PA14 infection, treatment with Xuebijing (25%–100%) noticeably inhibited the increase in the expression of these 3 antimicrobial genes (Figure 3). More importantly, after PA14 infection, treatment with 100% Xuebijing recovered the expression of these 3 antimicrobial genes to a control level (Figure 3).

### Xuebijing did not have obvious antibacterial activity

**FIGURE 4:**
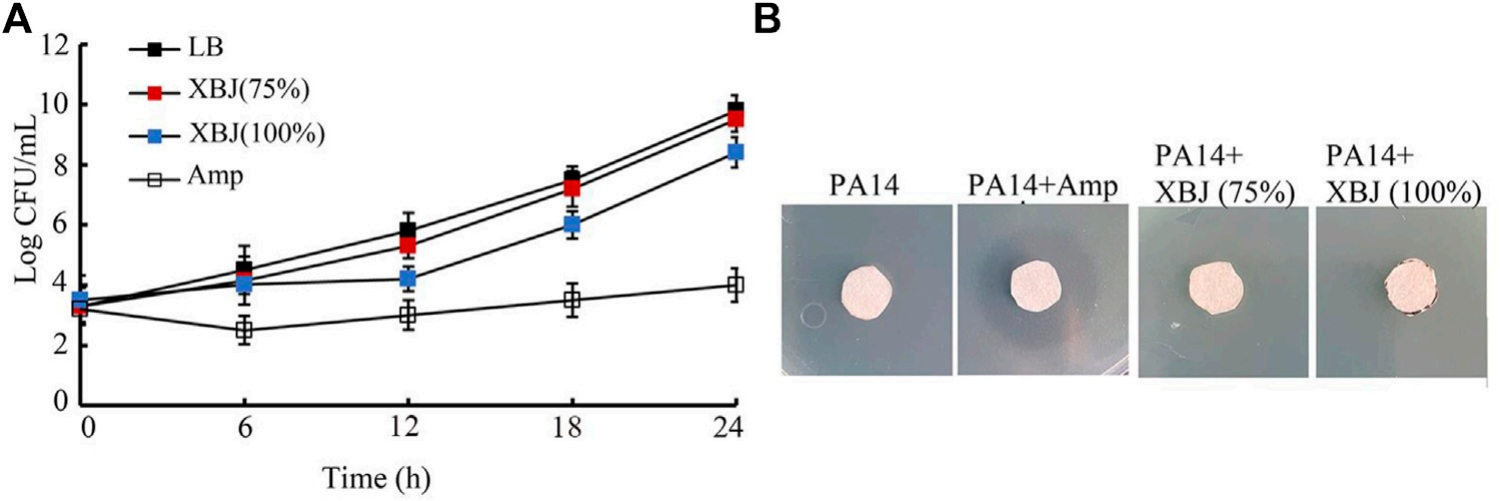
Analysis of the antibacterial activity of Xuebijing. **(A)** Time–kill assay. **(B)** Disk diffusion assay. XBJ, Xuebijing. AMP, ampicillin. AMP treatment concentration was 1 μg/mL.

In the time–kill assay, the 75% Xuebijing could not exhibit obvious antibacterial activity from 6 to 24 h (Figure 4A). The 100% Xuebijing treatment showed only moderate antibacterial activity from 9 to 24 h, which was very different from that of ampicillin (1 μg/mL) treatment (Figure 4A). Agar diffusion assay was further performed after 75% or 100% Xuebijing treatment. Compared with the noticeable zone of inhibition caused by 1 μg/mL ampicillin, both 75% Xuebijing and 100% Xuebijing could not result in an obvious zone of PA14 inhibition (Figure 4B).

### Effect of Xuebijing on biofilm formation and expression of virulence genes and quorum sensing-related genes of PA14

**FIGURE 5:**
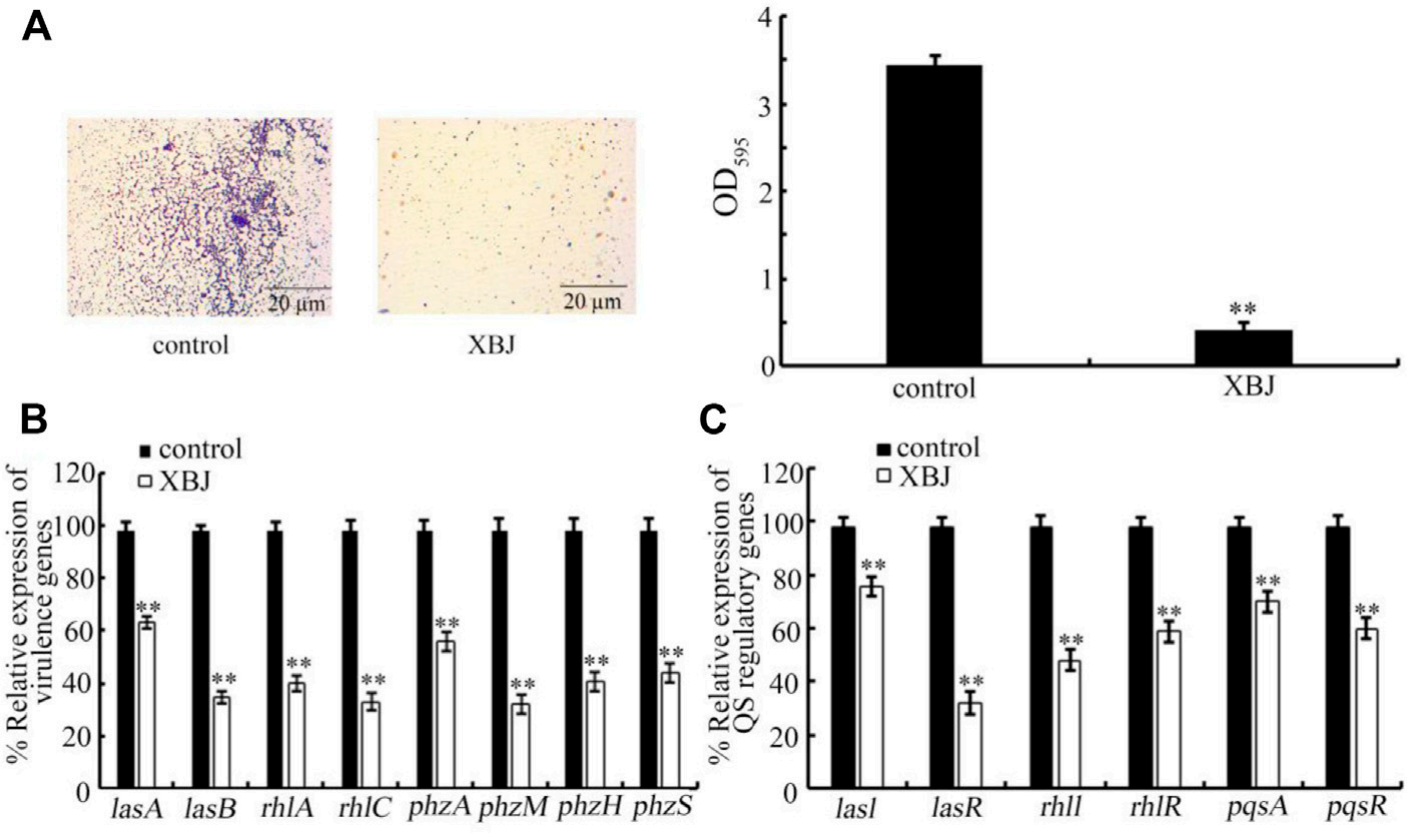
Effect of Xuebijing post-treatment on biofilm formation **(A)**, expression of virulence genes **(B)**, and expression of QS-related genes **(C)** of PA14. The 100% Xuebijing was used for pharmacological treatment. XBJ, Xuebijing. **p < 0.01 vs. control.

Biofilm formation is one of the important virulence factors for *P. aeruginosa* (Sharma et al., 2014; Skariyachan et al., 2018). Treatment with Xuebijing (100%) significantly inhibited the biofilm formation of PA14 (Figure 5A). In addition, Xuebijing post-treatment also affected the expression of virulence genes of PA14. After the Xuebijing (100%) treatment, the expression of virulence genes (*lasA*, *lasB*, *rhlA*, *rhlC*, *phzA*, *phzM*, *phzH*, and *phzS*) was significantly decreased (Figure 5B).

In bacteria, the quorum sensing (QS) mediates cell-to-cell communication mechanism to detect the community density (Miller and Bassler, 2001; Abisado et al., 2018). Moreover, we found that the expression of QS-related genes (*lasI*, *lasR*, *rhlI*, *rhlR*, *pqsA*, and *pqsR*) of PA14 was significantly decreased by Xuebijing (100%) treatment (Figure 5C).

### Beneficial effect of Xuebijing against bacterial infection depended on insulin, p38 MAPK, Wnt, DBL-1/TGF-β, ELT-2, and programmed cell death signals

**FIGURE 6:**
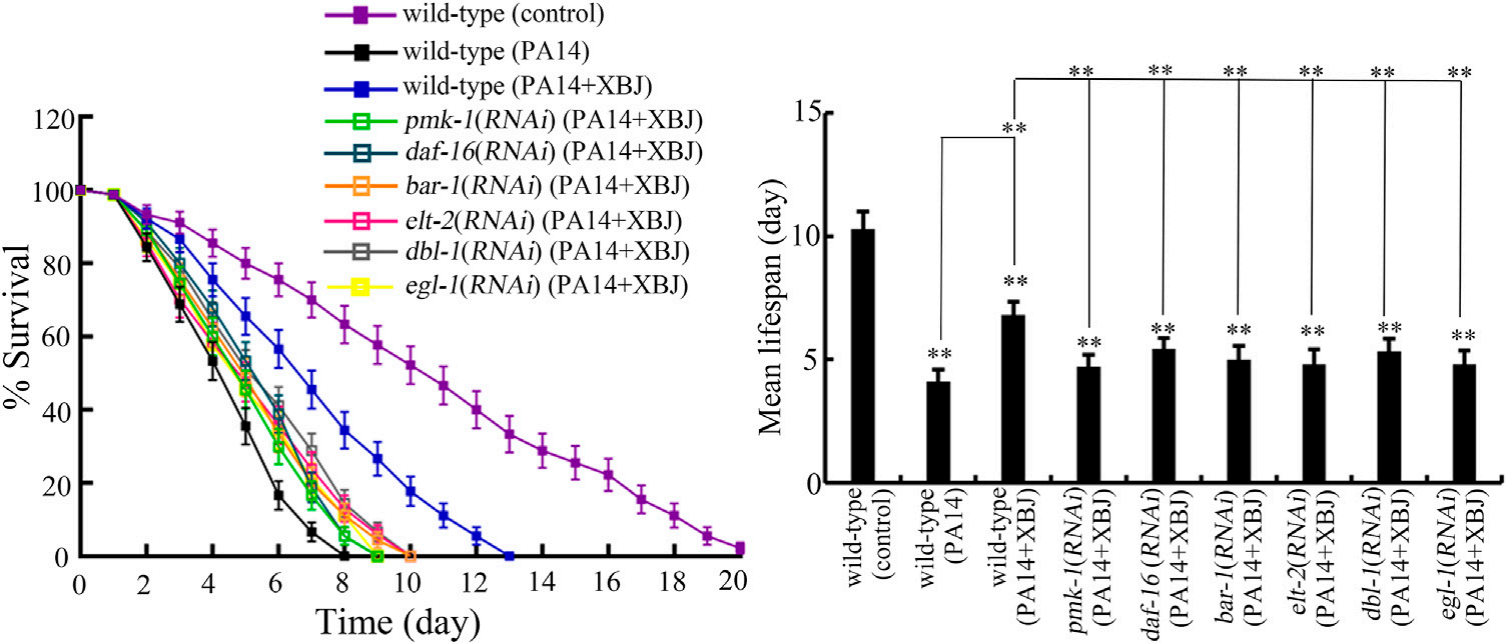
RNAi of *bar-1*, *elt-2*, *pmk-1*, *dbl-1*, *egl-1*, and *daf-16* altered the effect of Xuebijing (100%) treatment against *P*. *aeruginosa* infection. RNAi was performed after *P*. *aeruginosa* infection. XBJ, Xuebijing. **p < 0.01 vs. wild-type (control) (if not specially indicated).

Insulin, p38 MAPK, Wnt, DBL-1/TGF-β, ELT-2, and PCD are important signals required for the control of innate immunity against bacterial infection in nematodes (Kurz and Tan, 2004; Irazoqui et al., 2008; Arvanitis et al., 2013; Zhi et al., 2017b; Head et al., 2017). *pmk-1* encodes p38 MAPK in the p38 MAPK signaling pathway. *daf-16* and *bar-1* encode transcriptional factors of insulin and Wnt signaling pathways, respectively. *dbl-1* encodes a TGF-β ligand. *elt-2* encodes GATA transcriptional factor. *egl-1* encodes a BH3 protein in the PCD signaling pathway. After PA14 infection, the function of Xuebijing treatment against bacterial infection could be inhibited by RNAi of *pmk-1*, *daf-16*, *bar-1*, *dbl-1*, *egl-1*, and *elt-2* (Figure 6; Supplementary Table S4). RNAi of *pmk-1*, *daf-16*, *bar-1*, *dbl-1*, *egl-1*, and *elt-2* caused the similar inhibition on the beneficial function of Xuebijing treatment against PA14 infection (Figure 6; Supplementary Table S4). These observations suggested the involvement of these molecular signals in regulating the formation of the beneficial function for Xuebijing against bacterial infection.

In *C. elegans*, PA14 infection caused a significant decrease in expressions of *pmk-1*, *daf-16*, *bar-1*, *dbl-1*, *egl-1*, and *elt-2* (Supplementary Figure S3). Treatment with 100% Xuebijing suppressed this decrease in expressions of *pmk-1*, *daf-16*, *bar-1*, *dbl-1*, *egl-1*, and *elt-2* caused by PA14 infection (Supplementary Figure S3).

### Role of Xuebijing in reducing bacterial colony accumulation in the intestine was dependent on insulin, p38 MAPK, Wnt, DBL-1/TGF-β, ELT-2, and PCD signals

**FIGURE 7:**
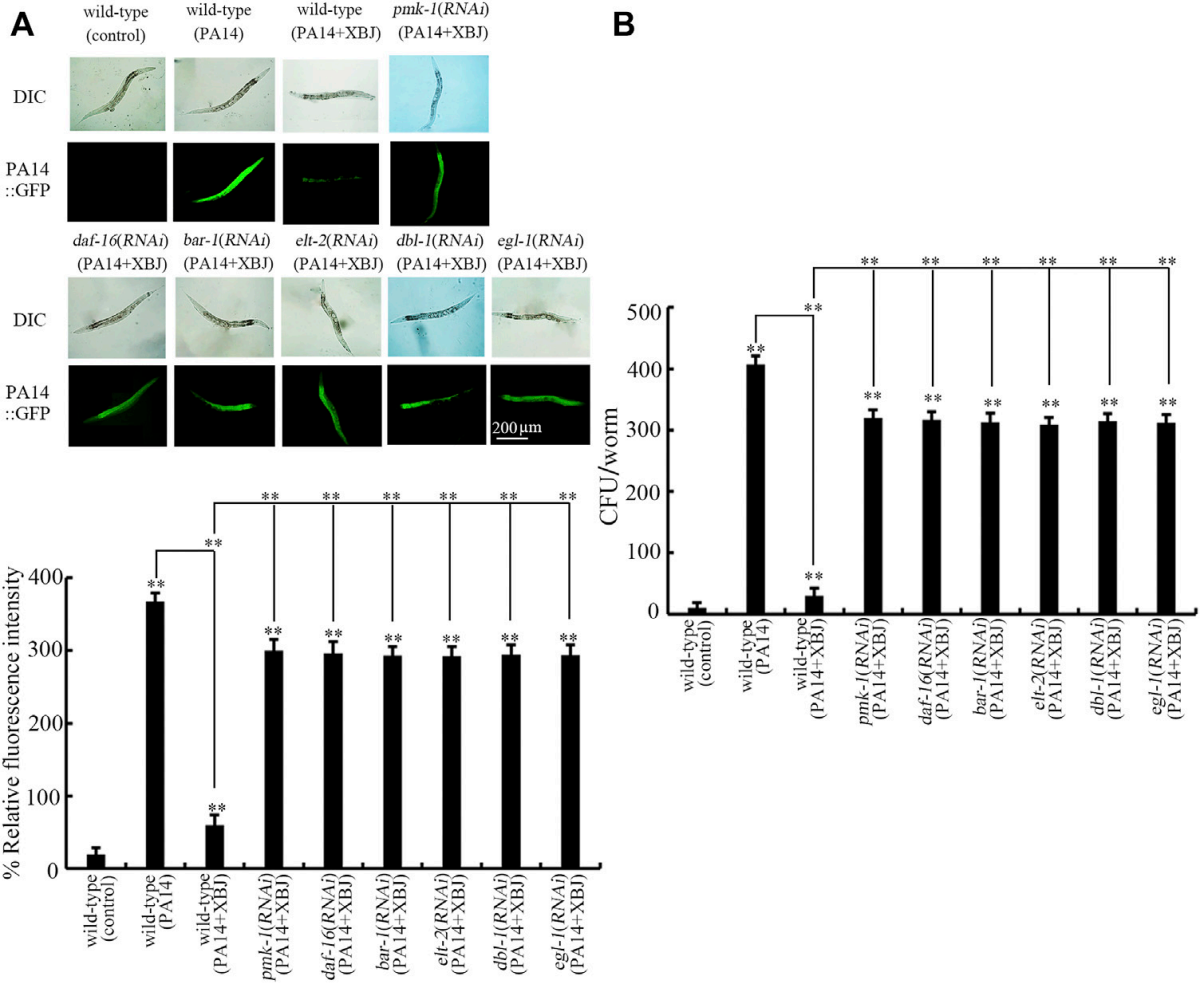
RNAi of *bar-1*, *elt-2*, *pmk-1*, *dbl-1*, *egl-1*, and *daf-16* affected the beneficial effect of Xuebijing (100%) treatment to reduce PA14:GFP **(A)** and to inhibit CFU **(B)** in *P*. *aeruginosa*-infected nematodes. RNAi was performed after *P*. *aeruginosa* infection. XBJ, Xuebijing. **p < 0.01 vs. wild-type (control) (if not specially indicated).

Besides the effect on Xuebijing treatment against bacterial infection, we also examined the effect of *pmk-1*, *daf-16*, *bar-1*, *dbl-1*, *egl-1*, and *elt-2* RNAi on PA14:GFP accumulation and bacterial CFU in the intestine after Xuebijing treatment. In nematodes with RNAi of *pmk-1*, *daf-16*, *bar-1*, *dbl-1*, *egl-1,* and *elt-2*, the function of Xuebijing treatment in inhibiting PA14 colony accumulation reflected by PA14::GFP and CFU was further obviously suppressed (Figures 7A,B).

### Effect of RNAi of *pmk-1*, *daf-*16, *bar-1*, and *elt-2* on the expression of their targeted genes in Xuebijing-treated nematodes after bacterial infection

In nematodes, the gamma-glutamylcysteine synthetase GCS-1 acted as a target of PMK-1 in p38 MAPK signaling pathway during the control of bacterial pathogenic resistance (Papp et al., 2012). The superoxide dismutase SOD-3, a downstream target of DAF-16 in the insulin signaling pathway, could be induced in the intestine after infection with pathogenic bacteria (Chávez et al., 2007). The homeobox gene *egl-5* acted downstream of β-catenin/*bar-1* to regulate the innate immune response to pathogenic infection (Irazoqui et al., 2008). An early immune response gene *F55G11.2* functioned as the target of *elt-2* in regulating the immune response to *P. aeruginosa* (Block et al., 2015). After PA14 infection, *gcs-1* expression was decreased by RNAi of *pmk-1* in Xuebijing-treated nematodes, *sod-3* expression was decreased by RNAi of *daf-16* in Xuebijing-treated nematodes, *egl-5* expression was decreased by RNAi of *bar-1* in Xuebijing-treated nematodes, and *F55G11.2* expression was decreased by RNAi of *elt-2* in Xuebijing-treated nematodes (Supplementary Figure S4).

## Discussion

In the organisms, including the humans, *P. aeruginosa* can break down biological barriers (Berube et al., 2016). Largely due to this, *Pseudomonas* infection is one of the important reasons for sepsis (Cheluvappa et al., 2009). The *Pseudomonas* sepsis has been frequently observed in the clinical ICU (Stone, 1966; Ho et al., 2020). Therefore, we selected *Pseudomonas* PA14 as an example of bacterial pathogens to determine the possible antibacterial infection property of Xuebijing on the host of *C. elegans*. *C. elegans* is an important animal model for innate immunity and stress response (Engelmann and Pujol, 2010; Liu et al., 2021; Wang et al., 2021; Zhao Y.-L. et al., 2022). After infection, Xuebijing treatment effectively inhibited the damage of PA14 infection in reducing lifespan, and this beneficial effect of Xuebijing was dose-dependent (Figure 1B). Our finding implies that the clinical Xuebijing administration may have beneficial effects to decrease the fatality rate caused by bacterial pathogenic infection. Nevertheless, the combinational use of Xuebijing with other drugs (such as certain antibiotics) is still suggested, since even treatment with 100% Xuebijing did not recover the lifespan of PA14-infected nematodes to the control level (Figure 1B). Under the normal condition, the 25%–100% Xuebijing treatment did not affect brood size and locomotion behavior and induce the obvious oxidative stress (data not shown). In addition, under the normal condition, the 25%–75% Xuebijing treatment did not affect lifespan, and the 100% Xuebijing treatment could even moderately increase lifespan (data not shown).

One of the important cellular mechanisms for antibacterial infection of Xuebijing was the reduction of pathogen colony formation in the intestinal lumen of nematodes. After infection, the accumulation of bacterial colony in the intestinal lumen is usually an important contributor to inducing and enhancing the damage of pathogen on nematodes (Aballay and Ausubel, 2002). Based on the CFU assay and PA14::GFP assessment, we observed that the colonization and accumulation of PA14 in the intestinal lumen were obviously reduced by Xuebijing treatment (Figure 2). Particularly, treatment with 100% Xuebijing could remove most of the PA14 from the intestinal lumen (Figure 2). These observations indicate that the Xuebijing treatment is useful for the excretion of bacterial pathogens from the digestive system in nematodes. Nevertheless, accompanied by the removal of most of PA14 from the intestinal lumen, we did not find the recovery of the lifespan curve to the control level after 100% Xuebijing treatment (Figure 1B). This implies that the Xuebijing post-treatment may have the limited effect to attenuate the damage on longevity already caused by PA14 infection.

In organisms, including the nematodes, antimicrobial peptides will be activated to kill pathogens after infection (Gravato-Nobre, 2005; Santaolalla et al., 2011). Modulation of innate immune response is also an important pharmacological mechanism for the examined drugs in nematodes (Shu et al., 2016). We further observed that the activation of antimicrobial genes by PA14 infection could be significantly suppressed by the following Xuebijing treatment (Figure 3). One of the reasons for this suppression of the activation of antimicrobial genes in *Pseudomonas*-infected nematodes may be due to the reduction and the excretion of bacterial pathogens from the intestinal lumen by Xuebijing treatment discussed above. That is, after Xuebijing treatment, the reduction in *Pseudomonas* cells from the intestinal lumen may gradually remove the requirement for nematodes to secret antimicrobial genes to kill bacterial pathogens.

For the molecular mechanism of Xuebijing treatment against bacterial infection, we found the requirement of insulin, p38 MAPK, Wnt, DBL-1/TGF-β, ELT-2, and PCD-related signals for the formation of the beneficial function of Xuebijing against bacterial infection. Under the condition of RNAi knockdown of *daf-16*, *pmk-1*, *bar-1*, *dbl-1*, *egl-1*, and *elt-2*, Xuebijing lacked the potential to increase the lifespan of PA14-infected nematodes (Figure 6; Supplementary Table S3). After PA14 infection, the expression of the corresponding targeted gene was inhibited by RNAi of *pmk-1*, *daf-16*, *bar-1*, or *elt-2* in Xuebijing-treated nematodes (Supplementary Figure S4), which further supported the role of these molecular signals in regulating the beneficial function of Xuebijing against bacterial infection. Meanwhile, we observed that the PA14-induced decrease in expressions of *pmk-1*, *daf-16*, *bar-1*, *dbl-1*, *egl-1*, and *elt-2* could be suppressed by 100% Xuebijing treatment (Supplementary Figure S3). These results suggest that Xuebijing treatment could inhibit the toxic effects caused by *Pseudomonas* infection by increasing the function of insulin, p38 MAPK, Wnt, DBL-1/TGF-β, ELT-2, and PCD-related signals. Nevertheless, we also noticed that, after 100% Xuebijing treatment, the expression of *pmk-1*, *daf-16*, *bar-1*, *dbl-1*, *egl-1*, and *elt-2* could not be recovered to control levels in PA14-infected nematodes (Supplementary Figure S3), which is consistent with the limited effect observed in Figure 1B. The reason to examine the effect of insulin, p38 MAPK, Wnt, DBL-1/TGF-β, ELT-2, and PCD-related signals is that these 6 molecular signals are crucial and conserved during the control of innate immunity and stress response (Kurz and Tan, 2004; Irazoqui et al., 2008; Arvanitis et al., 2013; Head et al., 2017; Wang, 2019; Harding and Ewbank, 2021). These 6 molecular signals are involved in the immunology of various diseases, such as diabetes, cancer, and hematologic disorders (Rossini et al., 1985; Gao et al., 2015; Kim and Choi, 2015; Batlle and Massague, 2019; Galluzzi et al., 2019; Bertheloot et al., 2021). In addition, some of them further directly participate in the control of immunity in humans. For example, the transcriptional factor GATA-3 is required for innate and adaptive immunity, and act as a master regulator for the differentiation of T helper (Th2) cells (Ho and Pai, 2007; Tindemans et al., 2014). Our results are useful to provide some clues to understand the underlying molecular mechanism of Xuebijing administration in the clinical ICU.

Moreover, we further found the requirement of insulin, p38 MAPK, Wnt, DBL-1/TGF-β, ELT-2, and PCD-related signals for the beneficial function of Xuebijing in inhibiting PA14 colony accumulation in the intestinal lumen (Figure 7). That is, the altered bacterial colony accumulation in the intestinal lumen is an important cellular mechanism for the function of *pmk-1*, *daf-16*, *bar-1*, *dbl-1*, *egl-1*, and *elt-2* in modulating the effect of Xuebijing against bacterial infection. This also suggested the biological function of insulin, p38 MAPK, Wnt, DBL-1/TGF-β, ELT-2, and PCD-related signals in modulating bacterial colonization in the intestine of nematodes. More importantly, this further supports the crucial role of Xuebijing in reducing bacterial colony accumulation in the body of nematodes.

Besides what we have discussed above, the direct antibacterial activity may also act as a crucial contributor to the formation of antibacterial infection property of Xuebijing treatment in nematodes. However, both time–kill assay and agar diffusion assay did not show the obvious antibacterial activity of Xuebijing (Figure 4). This implies that the 5 Chinese medicines and the compounds in these Chinese medicines may have no or only very limited antibacterial activity. The published references have also not implied the strong antibacterial activity of Xuebijing (Fan et al., 2020).

In this study, although we did not observe the obvious antibacterial activity for Xuebijing treatment, we found that the Xuebijing treatment could reduce the *Pseudomonas* virulence factors to a certain degree. For example, the biofilm formation of PA14 could be inhibited by Xuebijing (100%) treatment (Figure 5A). Meanwhile, both the expression of virulence genes (*lasA*, *lasB*, *rhlA*, *rhlC*, *phzA*, *phzM*, *phzH*, and *phzS*) and the expression of QS-related genes (*lasI*, *lasR*, *rhlI*, *rhlR*, *pqsA*, and *pqsR*) of PA14 were significantly decreased by Xuebijing (100%) treatment (Figures 5B,C). These observations suggest that, during the formation of antibacterial infection property, both the enhancement in the excretion of bacterial pathogens and the reduction in virulence factors may act as primary contributors to the beneficial effect of Xuebijing treatment against bacterial infection. Once the population density of bacteria reaches the critical threshold, the QS system will be activated to regulate both biofilm formation and expression of virulence genes (Warrier et al., 2021). Our data suggested that Xuebijing treatment also had the potential to suppress the QS mechanism in PA. The bioactive components in Chinese medicines in Xuebijing with the functions to enhance excreting bacterial pathogens out of the body of organisms and to reduce virulence factors are needed to be further carefully identified. A previous study has indicated that the paeonol, one of the active compounds in Xuebijing, inhibited the biofilm formation of Gram-negative bacteria, downregulated expressions of both QS-related genes and virulence genes of *P. aeruginosa*, and enhanced the survival rate of *C. elegans* (Yang D. et al., 2021). In addition, considering the pH value of Xuebijing injection is 5.5, the observed effect of Xuebijing on *P. aeruginosa* was not associated with the possible excessive high or low pH value of Xuebijing. Excessive high or low pH values will cause adverse effects on organisms, such as *C. elegans* (Wang, 2020).

## Conclusion

Together, using *C. elegans* as an animal model, we examined the potential of Xuebijing against bacterial infection and the underlying mechanism. In nematodes, Xuebijing treatment could inhibit the damage of *Pseudomonas* infection in reducing lifespan. The role of antibacterial infection for Xuebijing treatment was largely due to the suppression of *Pseudomonas* colonization and accumulation in the intestinal lumen and the influence on innate immune response in nematodes. In *C. elegans,* p38 MAPK, insulin, Wnt, DBL-1/TGF-β, ELT-2, and PCD-related signals were required for beneficial effects of Xuebijing against *Pseudomonas* infection and intestinal colony accumulation. Moreover, the reduction in virulence factors also acted as another primary contributor to the beneficial effect of Xuebijing treatment against bacterial infection. The findings support the potential role of Xuebijing administration against bacterial infection in organisms. Our results will be helpful for understanding the pharmacological mechanisms of Xuebijing treatment in clinical therapy.

## Data Availability

The original contributions presented in the study are included in the article/Supplementary Material, further inquiries can be directed to the corresponding authors.

## Abbreviations

ANOVA, analysis of variance; CFU, colony-forming unit; ICU; intensive care units; LB, Luria–Bertani; PCD, programmed cell death; QS, quorum sensing; TCM, traditional Chinese medicine; NGM, nematode growth medium; qRT-PCR, quantitative real-time polymerase chain reaction; RNAi, RNA interference; RP-HPLC, reverse-phase high-performance liquid chromatography.
