# Supplementary material for: Beneficial effect of Xuebijing against Pseudomonas aeruginosa infection in Caenorhabditis elegans
Source: Front Pharmacol. 2022 Aug 31;13:949608. doi: 10.3389/fphar.2022.949608 (PMC9470999; doi:10.3389/fphar.2022.949608)
Supplement: Supplementary file 1 [file DataSheet1.doc]

**Supporting Information:**


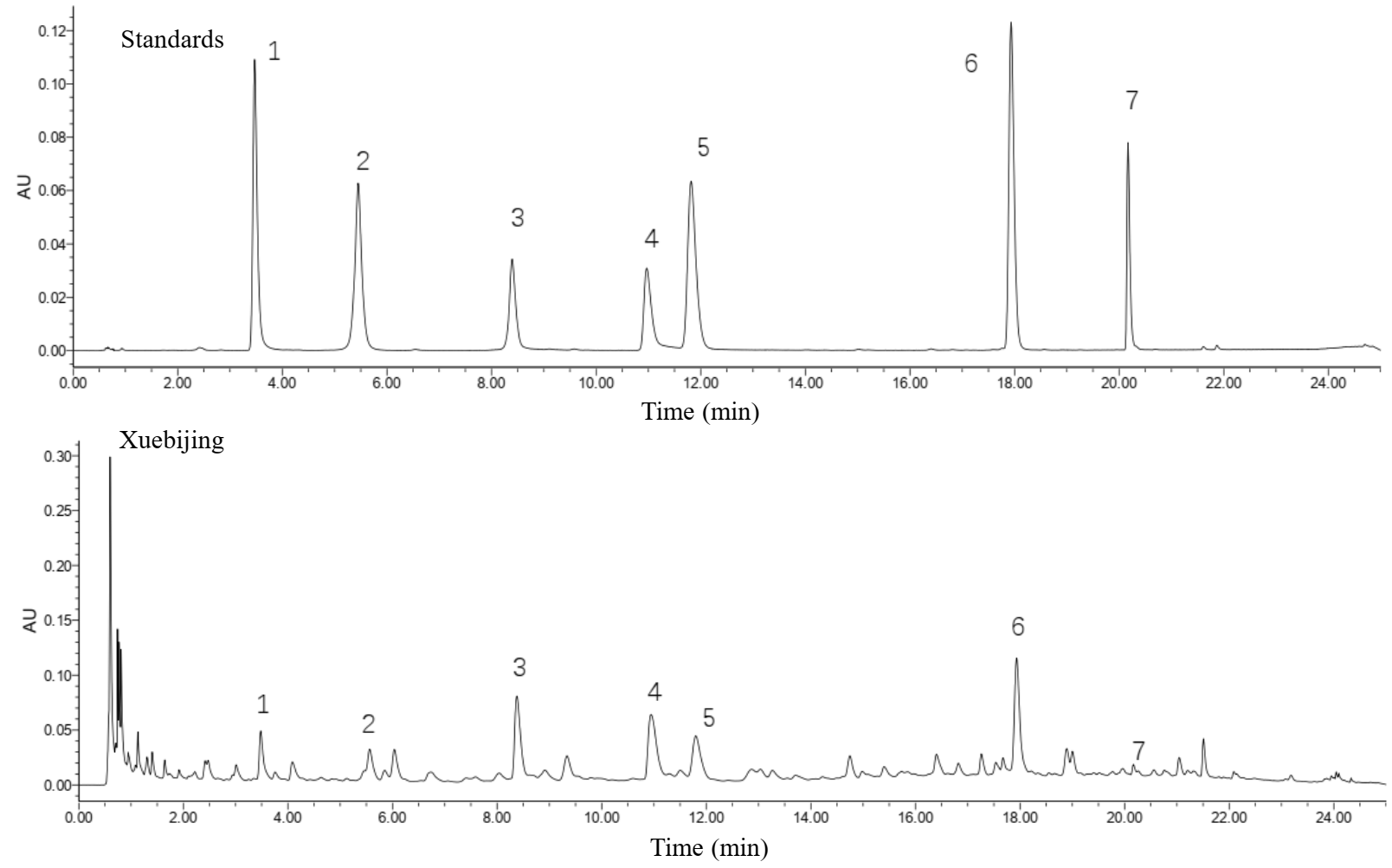


**Figure S1.** Biochemical fingerprint of Xuebijing. The fingerprint was analyzed by reverse phase high-performance liquid chromatography (RP-HPLC). The separation was performed on a Alltech C18 column with a moblie phase composed of 1% methanoic acid and [acetonitrile](https://dict.youdao.com/w/acetonitrile/#keyfrom=E2Ctranslation). The column temperature was set at 30℃ and the flow rate was 1.0 mL/min. The detective wavelength was set 280 nm. The injection volume was 10 μL. 1, Protocatechuic aldehyde; 2. Chlorogenic acid; 3. Hydroxysafflor yellow A; 4. Paeoniflorin; 5. Ferulic acid; 6. Senkyunolide; and 7. Salvianolic acid B.


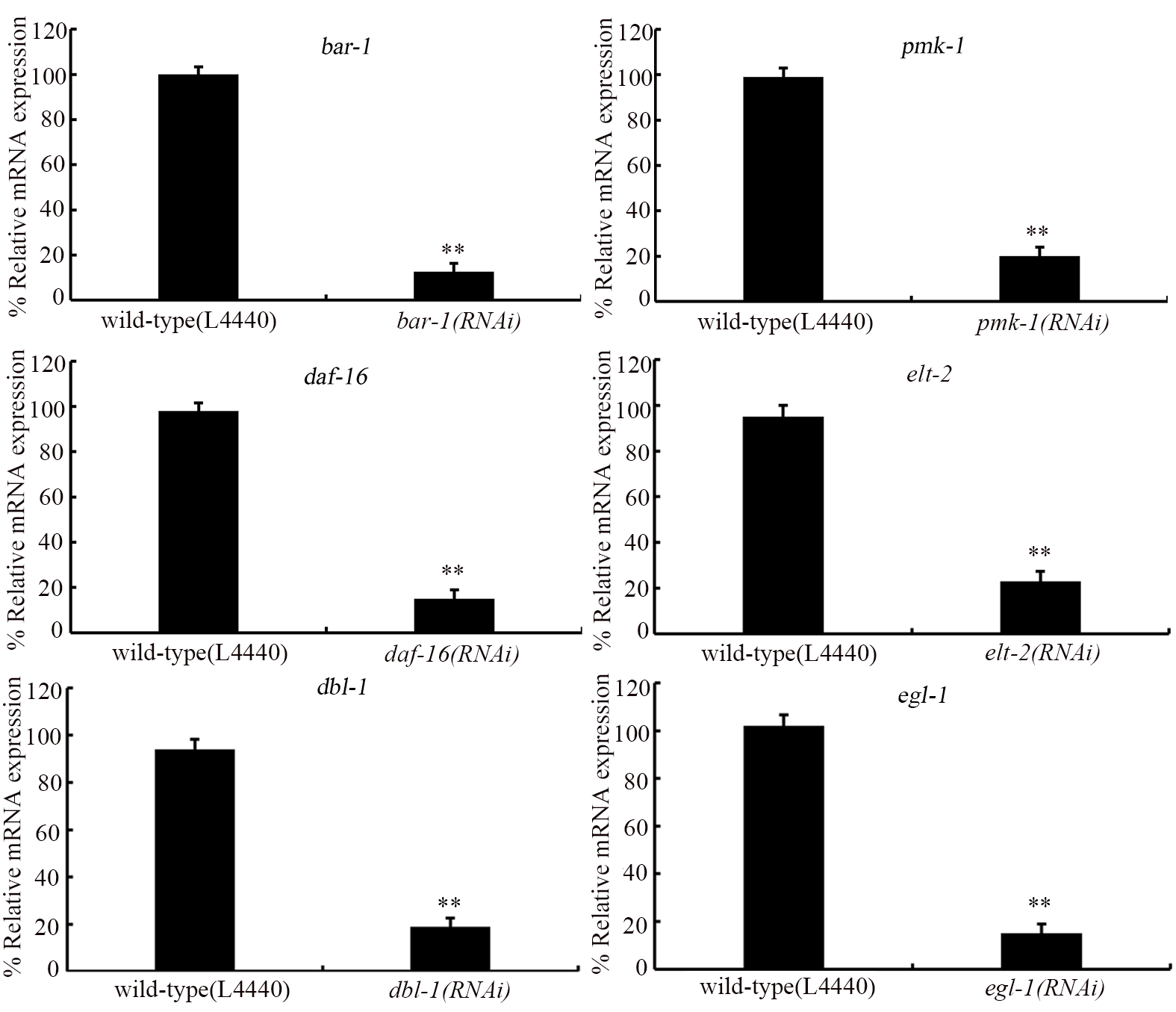


**Figure S2.** Efficiency of RNAi knockdown of *bar-1*, *pmk-1*, *daf-16*, *bar-1*, *egl-1*, and *elt-2* in wild-type nematodes. ^**^*P <* 0.01 *vs* wild-type(L4440).


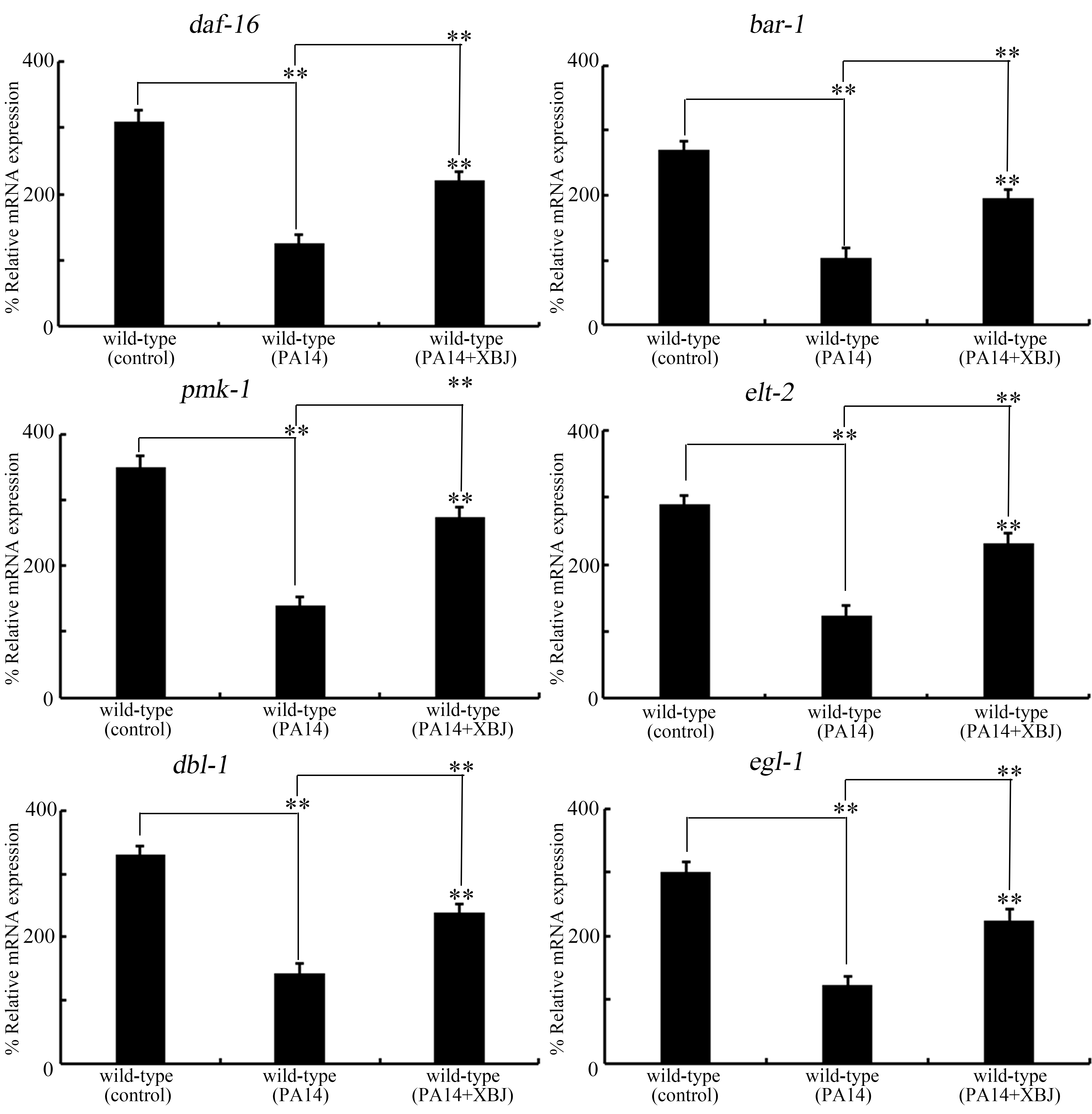


**Figure S3.** Effect of 100% Xuebijing treatment on expressions of *pmk-1*, *daf-16*, *bar-1*, *dbl-1*, *egl-1*, and *elt-2* in nematode after PA14 infection. XBJ, Xuebijing. ^**^*P <* 0.01.


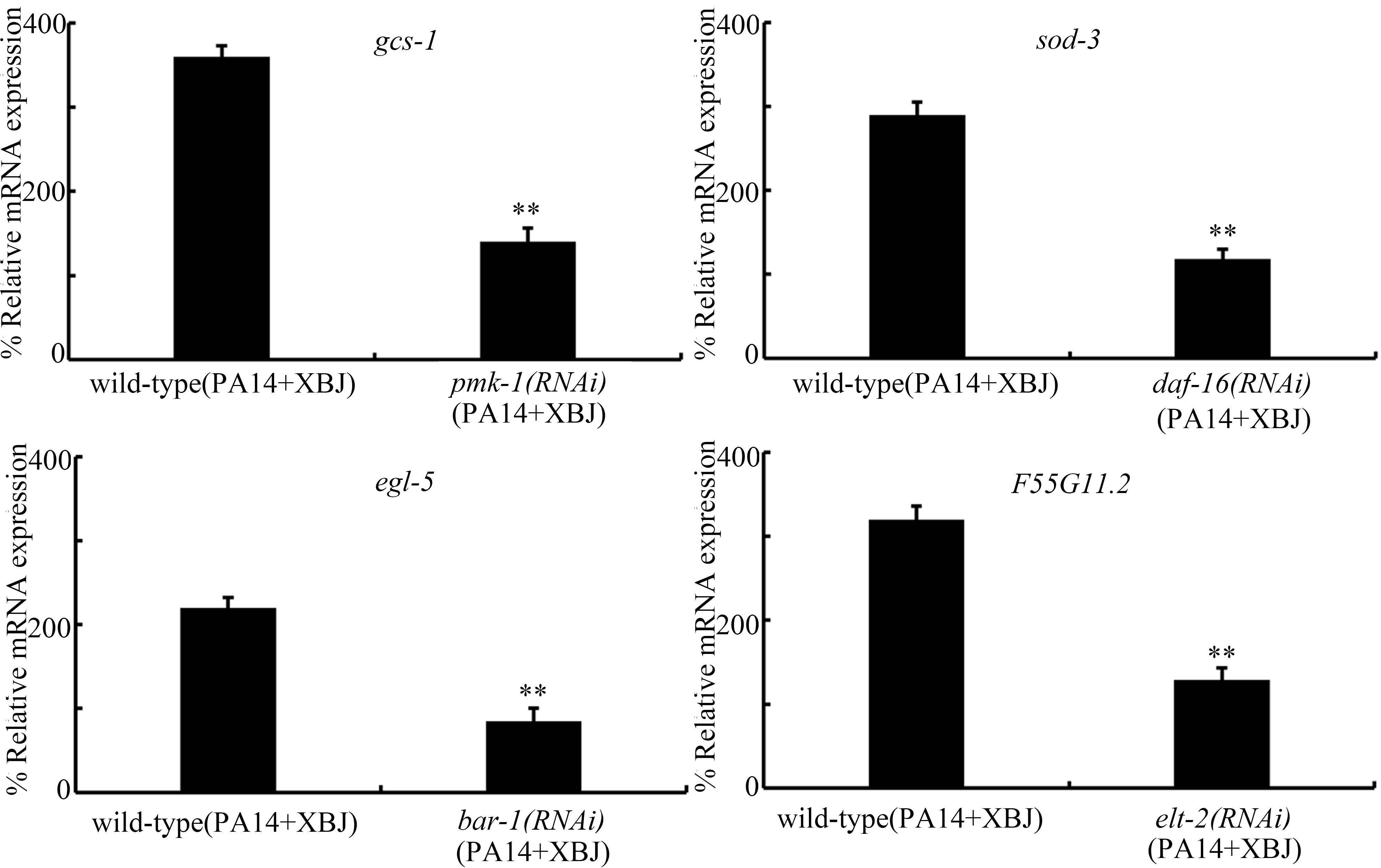


**Figure S4.** Effect of RNAi of *pmk-1*, *daf-16*, *bar-1*, and *elt-2* on expression of their downstream targets in Xuebijing treated nematodes after PA infection. XBJ, Xuebijing. ^**^*P <* 0.01 *vs* wild-type(PA14+XBJ).

**Table S1.** Primer information for qRT-PCR in *C. elegans*

| Gene | Forward primer (5’-3’) | Reverse primer (5’-3’) |
| --- | --- | --- |
| *lys-1* | CGAGCCAAGCAATACGGACT | ACCACCTCCAAGAACATGCC |
| *dod-22* | TCAGCTTCTGCATCCGTTTG | CAGCTGCACGTTTGTCCTTT |
| *lys-8* | ATTCGGCTTCAGTCTCCGTC | ACCAGCTTGTTTGAGGCACT |
| *gcs-1* | GCCTTACGGAGGTCTCATCG | TCCACGGAAGATTGGTGTGG |
| *sod-3* | ATCACTATTGCGGTTCAAGGCTCT | AATTCCAAAAAGTGGGACCATTCC |
| *egl-5* | ACGGTGAGTTGTTCGCATCA | CGGTGGACACAACGGGTATT |
| *F55G11.2* | TCCTCAGTTGGAAAAGGGGC | GCGTTGACAACCAGTCCAGT |
| *pmk-1* | TCCGACTCCACGAGAAGGAT | CACGATATGTACGACGGGCA |
| *daf-16* | ACCGTTGGTCAAATGCTTGC | TGGCTTCTTACGACAACGCT |
| *bar-1* | CCTAATTTGCACGCTACGGC | TCGGCATGATCGGAATGGAG |
| *dbl-1* | TTTTGCGGCGAACAAATCGT | TTCGCTGTTGCCTGTTTGTG |
| *egl-1* | GCCTCAACCTCTTCGGATCT | GCACATTGCTGCTAGCTTGG |
| *elt-2* | CTGCGCCTACCAACTCAGAA | GCCGTTCAATTGGAGATGCG |
| *tba-1* | TCAACACTGCCATCGCCGCC | TCCAAGCGAGACCAGGCTTCAG |

**Table S2.** Primer information for qRT-PCR in PA14

| Gene | Forward primer (5’-3’) | Reverse primer (5’-3’) |
| --- | --- | --- |
| *lasl* | CGCACATCTGGGAACTCA | CGGCACGGATCATCATCT |
| *lasR* | CTGTGGATGCTCAAGGACTAC | AACTGGTCTTGCCGATGG |
| *rhll* | GTAGCGGGTTTGCGGATG | CGGCATCAGGTCTTCATCG |
| *rhlR* | GCCAGCGTCTTGTTCGG | CGGTCTGCCTGAGCCATC |
| *pqsA* | GACCGGCTGTATTCGATTC | GCTGAACCAGGGAAAGAAC |
| *pqsR* | CTGATCTGCCGGTAATTGG | ATCGACGAGGAACTGAAGA |
| *lasA* | CTGTGGATGCTCAAGGACTAC | AACTGGTCTTGCCGATGG |
| *lasB* | AACCGTGCGTTCTACCTGTT | CGGTCCAGTAGTAGCGGTTG |
| *rhlA* | TGGCCGAACATTTCAACGT | GATTTCCACCTCGTCGTCCTT |
| *rhlC* | GCCATCCATCTCGACGGAC | CGCAGGCTGTATTCGGTG |
| *phzA* | AACGGTCAGCGGTACAGGGAAC | AACGGTCAGCGGTACAGGGAAAC |
| *phzM* | ACGGCTGTGGCGGTTTA | CCGTGACCGTCGCATT |
| *phzH* | GCTCATCGACAATGCCGAACT | GCGGATCTCGCCGAACATCAG |
| *phzS* | CCGAAGGCAAGTCGCTGGTGA | GGTCCCAGTCGGCGAAGAACG |
| *pvdQ* | GCCGAGGAGATCGTCACC | CAGGCGTAGAAGATGTCGGA |

**Table S3.** Effect of Xuebijing post-treatment on lifespan of nematodes after *P*. *aeruginosa* infection

| Treatment | Mean lifespan  (day) | Number | *P <* 0.01 *vs* control | *P <* 0.01 *vs* PA14 |
| --- | --- | --- | --- | --- |
| control | 12.1 ± 0.62 | 50 |  |  |
| PA14 | 4.4 ± 0.51 | 50 | *P <* 0.01 |  |
| PA14+XBJ(25%) | 5.3 ± 0.62 | 50 | *P <* 0.01 |  |
| PA14+XBJ(50%) | 6.2 ± 0.71 | 50 | *P <* 0.01 | *P <* 0.01 |
| PA14+XBJ(75%) | 7.9 ± 0.57 | 50 | *P <* 0.01 | *P <* 0.01 |
| PA14+XBJ(100%) | 8.7 ± 0.56 | 50 | *P <* 0.01 | *P <* 0.01 |

XBJ, Xuebijing.

**Table S4.** Effect of *bar-1*, *elt-2*, *pmk-1*, *dbl-1*, *egl-1*, and *daf-16* RNAi on the role of Xuebijing (100%) treatment against *P*. *aeruginosa* infection

| Treatment | Mean lifespan  (day) | Number | *P <* 0.01 *vs* wild-type  (control) | *P <* 0.01 *vs* wild-type  (PA14) | *P <* 0.01 *vs* wild-type  (PA14+XBJ) |
| --- | --- | --- | --- | --- | --- |
| wild-type  (control) | 10 ± 0.71 | 50 |  |  |  |
| wild-type  (PA14) | 4.1 ± 0.52 | 50 | *P <* 0.01 |  |  |
| wild-type  (PA14+XBJ) | 6.8 ± 0.53 | 50 | *P <* 0.01 | *P <* 0.01 |  |
| *daf-16(RNAi)*  (PA14+XBJ) | 5.43 ± 0.45 | 50 | *P <* 0.01 |  | *P <* 0.01 |
| *pmk-1(RNAi)*  (PA14+XBJ) | 4.7 ± 0.5 | 50 | *P <* 0.01 |  | *P <* 0.01 |
| *bar-1(RNAi)*  (PA14+XBJ) | 5 ± 0.56 | 50 | *P <* 0.01 |  | *P <* 0.01 |
| *elt-2(RNAi)*  (PA14+XBJ) | 4.8 ± 0.61 | 50 | *P <* 0.01 |  | *P <* 0.01 |
| *dbl-1(RNAi)*  (PA14+XBJ) | 5.33 ± 0.51 | 50 | *P <* 0.01 |  | *P <* 0.01 |
| *egl-1(RNAi)*  (PA14+XBJ) | 4.82 ± 0.56 | 50 | *P <* 0.01 |  | *P <* 0.01 |

XBJ, Xuebijing.
